# Supplementary figures and images for: Direct addition of poly-lysine or poly-ethylenimine to the medium: A simple alternative to plate pre-coating
Source: PLoS One. 2022 Jul 8;17(7):e0260173. doi: 10.1371/journal.pone.0260173 (PMC9269970; doi:10.1371/journal.pone.0260173)

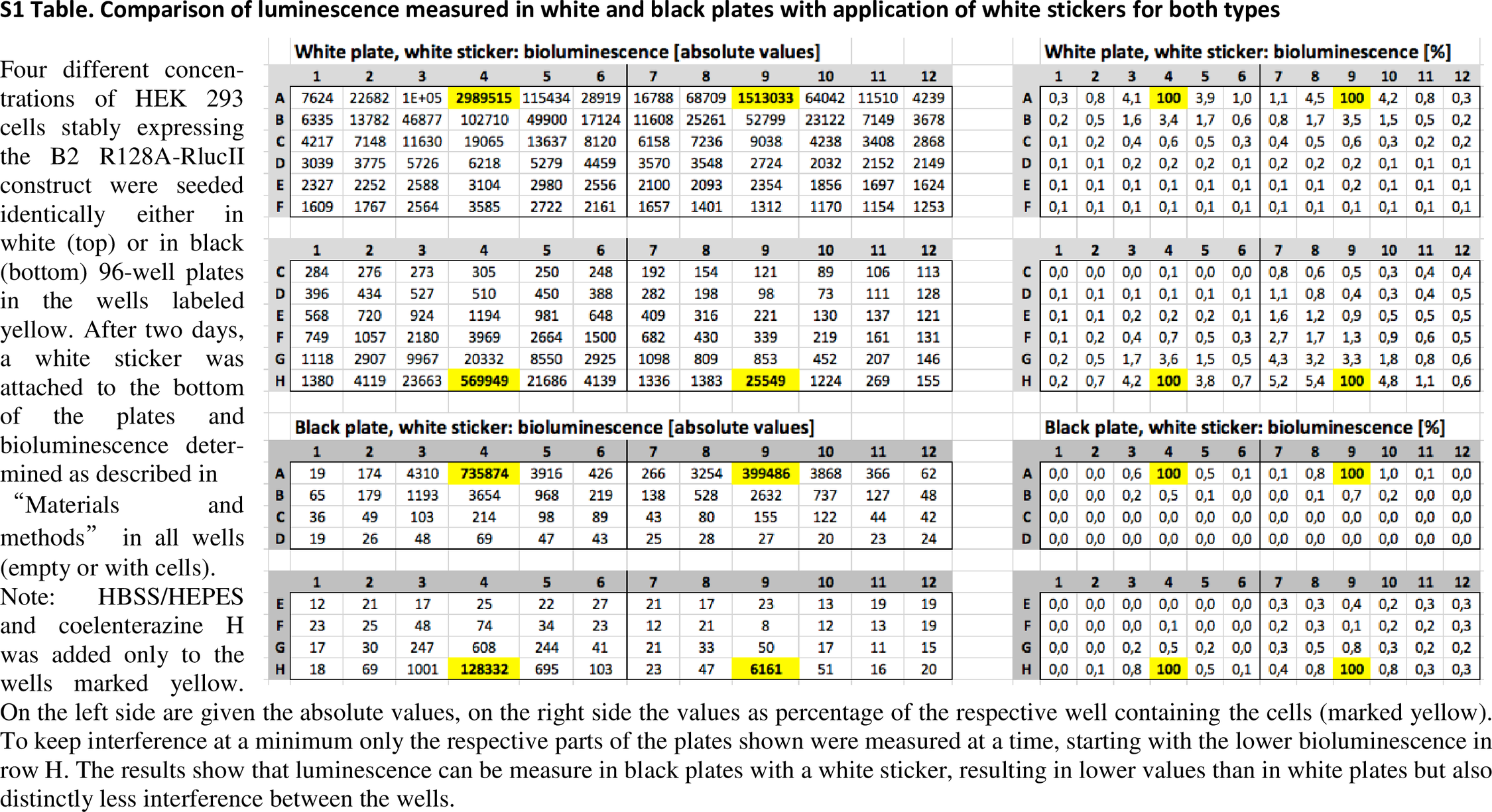

Supplement: S1 Table — Four different concentrations of HEK 293 cells stably expressing the B2 R128A-RlucII construct were seeded identically either in white (top) or in black (bottom) 96-well plates in the wells labeled yellow. After two days, a white sticker was attached to the bottom of the plates and bioluminescence determined as described in “Materials and methods” in all wells (empty or with cells). Note: HBSS/HEPES and coelenterazine H was added only to the wells marked yellow. On the left side are given the absolute values, on the right side the values as percentage of the respective well containing the cells (marked yellow). To keep interference at a minimum only the respective parts of the plates shown were measured at a time, starting with the lower bioluminescence in row H. The results show that luminescence can be measure in black plates with a white sticker, resulting in lower values than in white plates but also distinctly less interference between the wells. (TIF) [file pone.0260173.s001.tif]

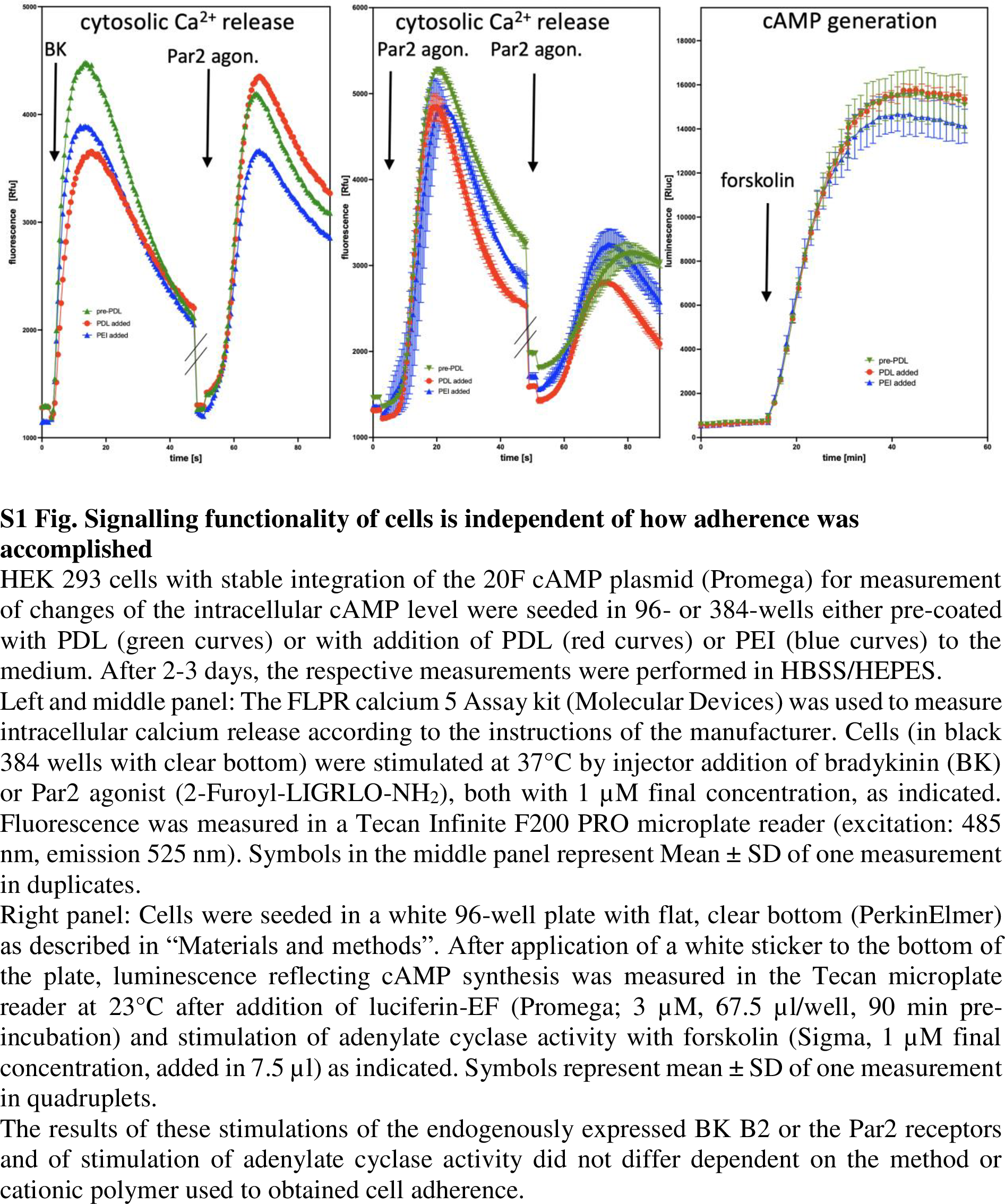

Supplement: S1 Fig — HEK 293 cells with stable integration of the 20F cAMP plasmid (Promega) for measurement of changes of the intracellular cAMP level were seeded in 96- or 384-wells either pre-coated with PDL (green curves) or with addition of PDL (red curves) or PEI (blue curves) to the medium. After 2–3 days, the respective measurements were performed in HBSS/HEPES. Left and middle panel: The FLPR calcium 5 Assay kit (Molecular Devices) was used to measure intracellular calcium release according to the instructions of the manufacturer. Cells (in black 384 wells with clear bottom) were stimulated at 37°C by injector addition of bradykinin (BK) or Par2 agonist (2-Furoyl-LIGRLO-NH2), both with 1 μM final concentration, as indicated. Fluorescence was measured in a Tecan Infinite F200 PRO microplate reader (excitation: 485 nm, emission 525 nm). Symbols in the middle panel represent Mean ± SD of one measurement in duplicates. Right panel: Cells were seeded in a white 96-well plate with flat, clear bottom (PerkinElmer) as described in “Materials and methods”. After application of a white sticker to the bottom of the plate, luminescence reflecting cAMP synthesis was measured in the Tecan microplate reader at 23°C after addition of luciferin-EF (Promega; 3 μM, 67.5 μl/well, 90 min pre-incubation) and stimulation of adenylate cyclase activity with forskolin (Sigma, 1 μM final concentration, added in 7.5 μl) as indicated. Symbols represent mean ± SD of one measurement in quadruplets. The results of these stimulations of the endogenously expressed BK B2 or the Par2 receptors and of stimulation of adenylate cyclase activity did not differ dependent on the method or cationic polymer used to obtained cell adherence. (TIF) [file pone.0260173.s002.tif]

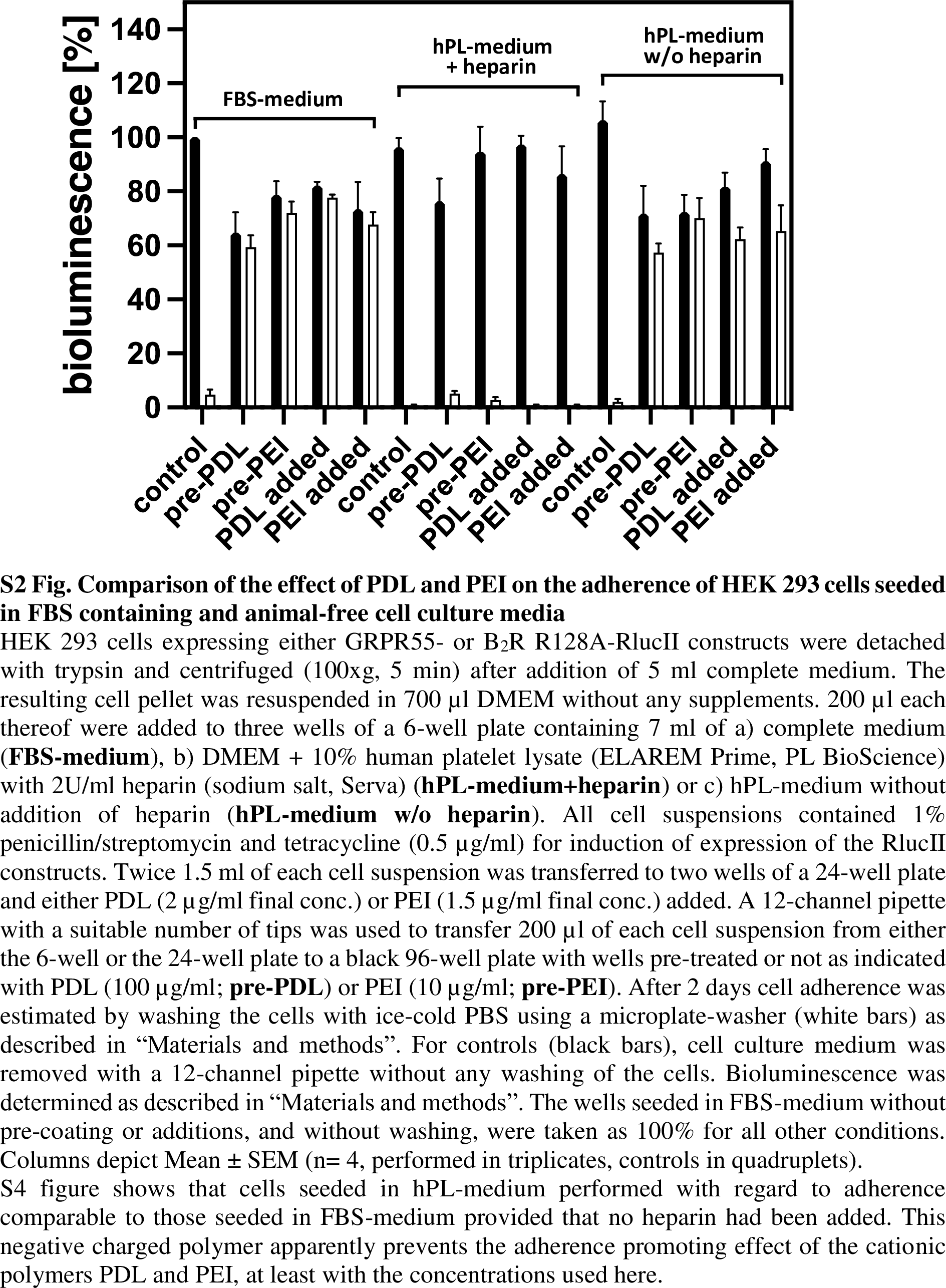

Supplement: S2 Fig — HEK 293 cells expressing either GRPR55- or B2R R128A-RlucII constructs were detached with trypsin and centrifuged (100xg, 5 min) after addition of 5 ml complete medium. The resulting cell pellet was resuspended in 700 μl DMEM without any supplements. 200 μl each thereof were added to three wells of a 6-well plate containing 7 ml of a) complete medium (FBS-medium), b) DMEM + 10% human platelet lysate (ELAREM Prime, PL BioScience) with 2U/ml heparin (sodium salt, Serva) (hPL-medium+heparin) or c) hPL-medium without addition of heparin (hPL-medium w/o heparin). All cell suspensions contained 1% penicillin/streptomycin and tetracycline (0.5 μg/ml) for induction of expression of the RlucII constructs. Twice 1.5 ml of each cell suspension was transferred to two wells of a 24-well plate and either PDL (2 μg/ml final conc.) or PEI (1.5 μg/ml final conc.) added. A 12-channel pipette with a suitable number of tips was used to transfer 200 μl of each cell suspension from either the 6-well or the 24-well plate to a black 96-well plate with wells pre-treated or not as indicated with PDL (100 μg/ml; pre-PDL) or PEI (10 μg/ml; pre-PEI). After 2 days cell adherence was estimated by washing the cells with ice-cold PBS using a microplate-washer (white bars) as described in “Materials and methods”. For controls (black bars), cell culture medium was removed with a 12-channel pipette without any washing of the cells. Bioluminescence was determined as described in “Materials and methods”. The wells seeded in FBS-medium without pre-coating or additions, and without washing, were taken as 100% for all other conditions. Columns depict Mean ± SEM (n = 4, performed in triplicates, controls in quadruplets). S4 Fig shows that cells seeded in hPL-medium performed with regard to adherence comparable to those seeded in FBS-medium provided that no heparin had been added. This negative charged polymer apparently prevents the adherence promoting effect of the cationic polymers PDL and PEI, at least w [file pone.0260173.s003.tif]

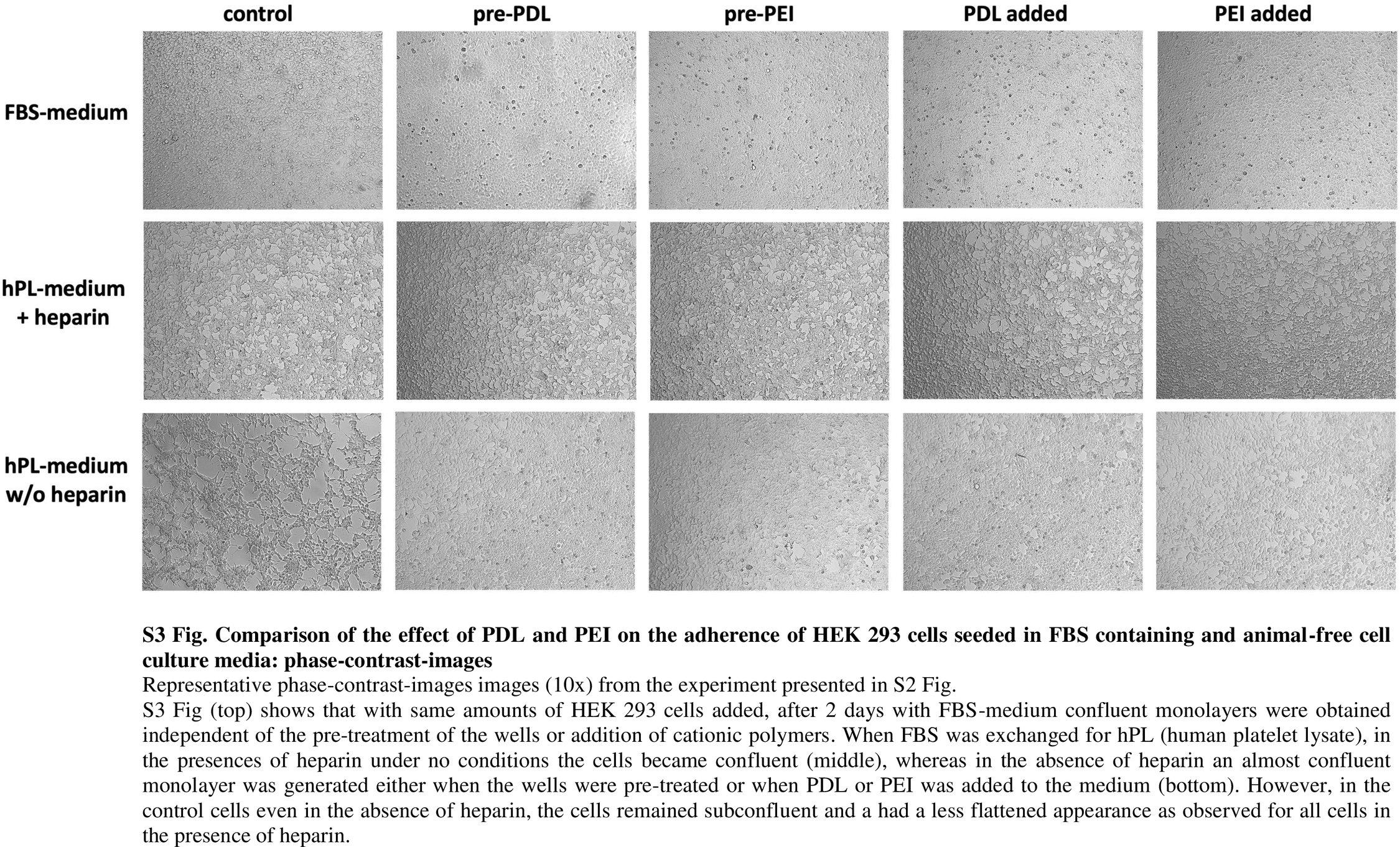

Supplement: S3 Fig — Representative phase-contrast-images images (10x) from the experiment presented in S2 Fig. S3 Fig (top) shows that with same amounts of HEK 293 cells added, after 2 days with FBS-medium confluent monolayers were obtained independent of the pre-treatment of the wells or addition of cationic polymers. When FBS was exchanged for hPL (human platelet lysate), in the presences of heparin under no conditions the cells became confluent (middle), whereas in the absence of heparin an almost confluent monolayer was generated either when the wells were pre-treated or when PDL or PEI was added to the medium (bottom). However, in the control cells even in the absence of heparin, the cells remained subconfluent and a had a less flattened appearance as observed for all cells in the presence of heparin. (TIF) [file pone.0260173.s004.tif]

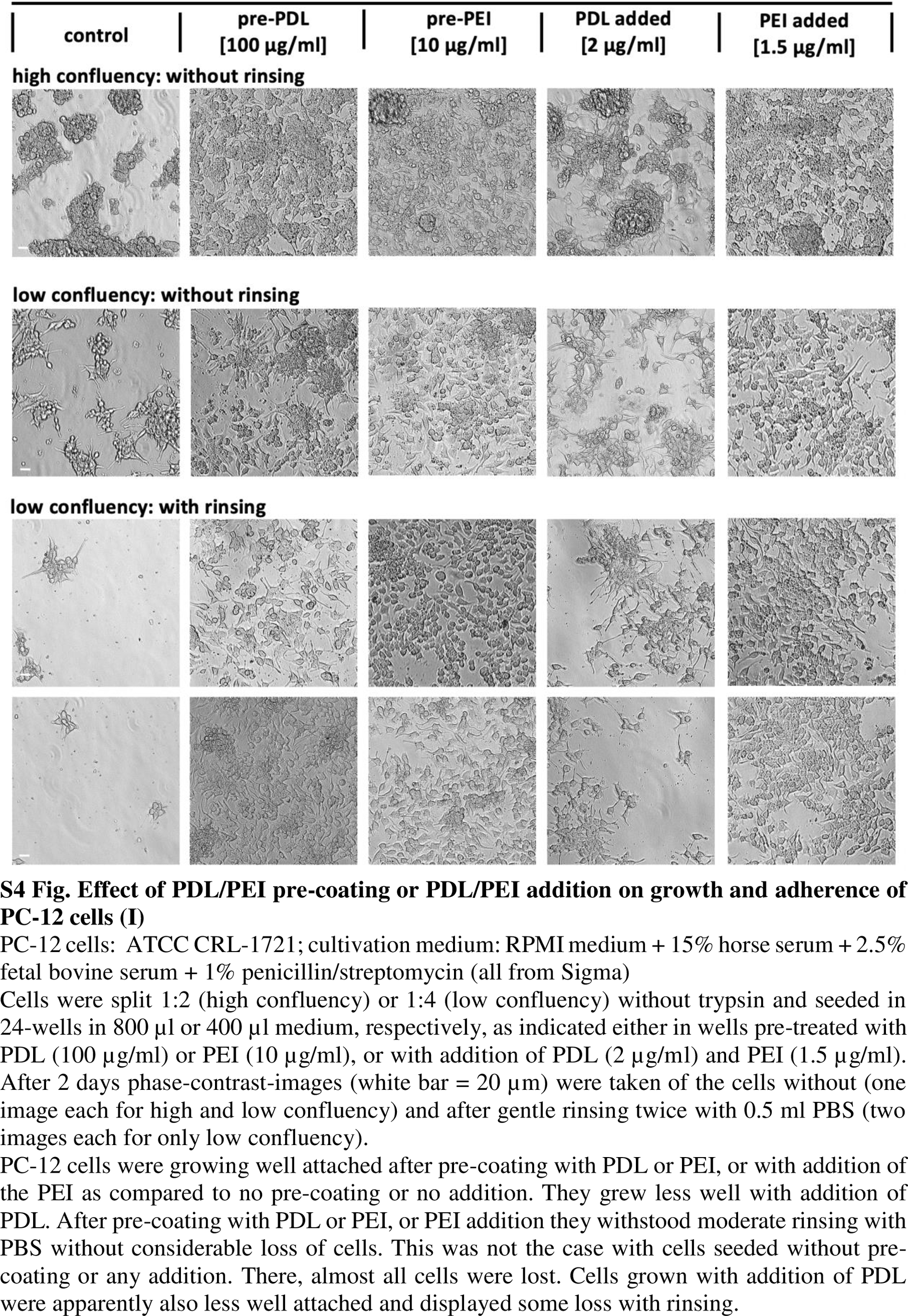

Supplement: S4 Fig — PC-12 cells: ATCC CRL-1721. Cultivation medium: RPMI medium + 15% horse serum + 2.5% fetal bovine serum + 1% penicillin/streptomycin (all from Sigma). Cells were split 1:2 (high confluency) or 1:4 (low confluency) without trypsin and seeded in 24-wells in 800 μl or 400 μl medium, respectively, as indicated either in wells pre-treated with PDL (100 μg/ml) or PEI (10 μg/ml), or with addition of PDL (2 μg/ml) and PEI (1.5 μg/ml). After 2 days phase-contrast-images (white bar = 20 μm) were taken of the cells without (one image each for high and low confluency) and after gentle rinsing twice with 0.5 ml PBS (two images each for only low confluency). PC-12 cells were growing well attached after pre-coating with PDL or PEI, or with addition of the PEI as compared to no pre-coating or no addition. They grew less well with addition of PDL. After pre-coating with PDL or PEI, or PEI addition they withstood moderate rinsing with PBS without considerable loss of cells. This was not the case with cells seeded without pre-coating or any addition. There, almost all cells were lost. Cells grown with addition of PDL were apparently also less well attached and displayed some loss with rinsing. (TIF) [file pone.0260173.s005.tif]

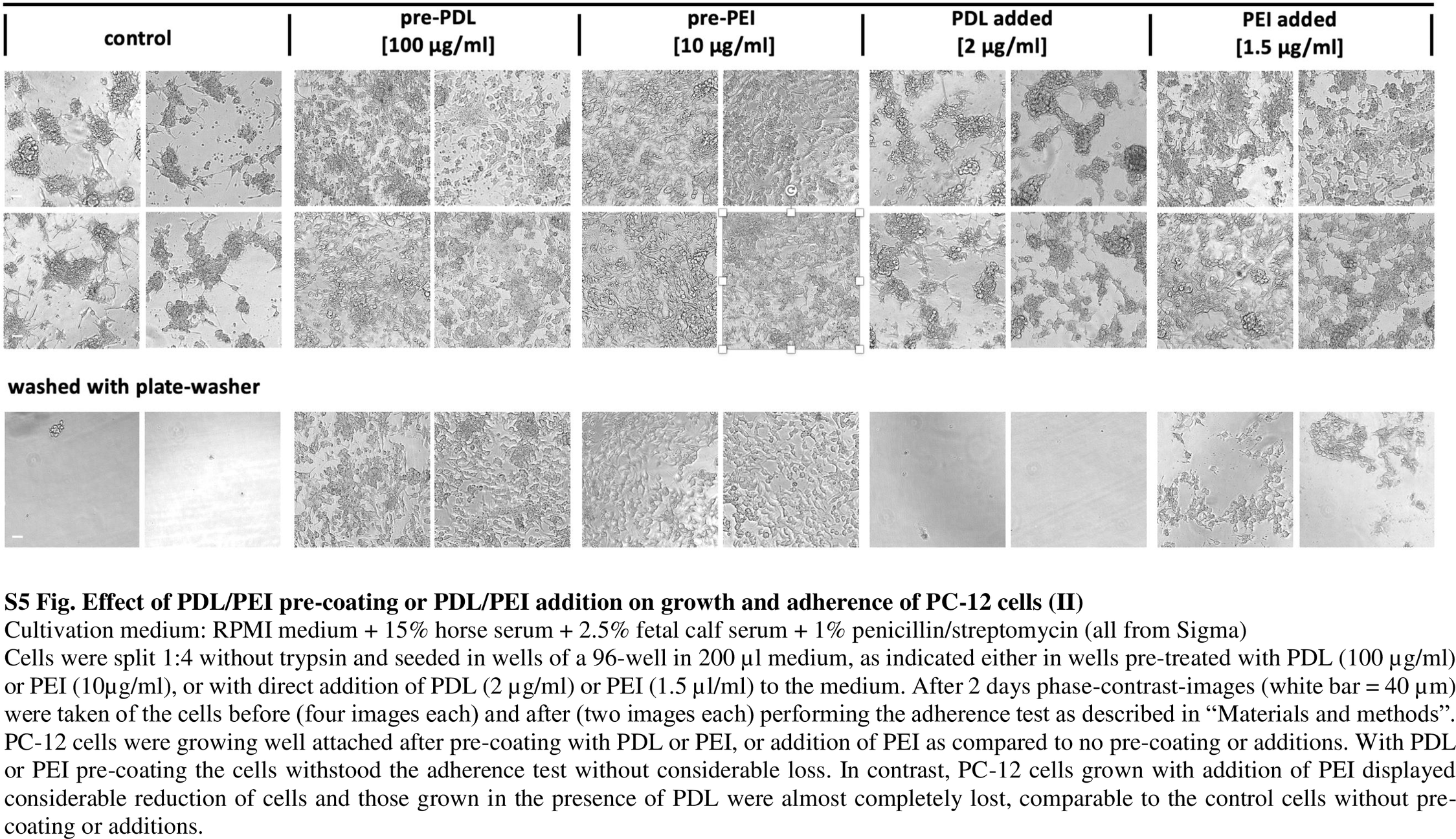

Supplement: S5 Fig — Cultivation medium: RPMI medium + 15% horse serum + 2.5% fetal calf serum + 1% penicillin/streptomycin (all from Sigma). Cells were split 1:4 without trypsin and seeded in wells of a 96-well in 200 μl medium, as indicated either in wells pre-treated with PDL (100 μg/ml) or PEI (10μg/ml), or with direct addition of PDL (2 μg/ml) or PEI (1.5 μl/ml) to the medium. After 2 days phase-contrast-images (white bar = 40 μm) were taken of the cells before (four images each) and after (two images each) performing the adherence test as described in “Materials and methods”. PC-12 cells were growing well attached after pre-coating with PDL or PEI, or addition of PEI as compared to no pre-coating or additions. With PDL or PEI pre-coating the cells withstood the adherence test without considerable loss. In contrast, PC-12 cells grown with addition of PEI displayed considerable reduction of cells and those grown in the presence of PDL were almost completely lost, comparable to the control cells without pre-coating or additions. (TIF) [file pone.0260173.s006.tif]
