# Supplementary material for: Direct addition of poly-lysine or poly-ethylenimine to the medium: A simple alternative to plate pre-coating
Source: PLoS One. 2022 Jul 8;17(7):e0260173. doi: 10.1371/journal.pone.0260173 (PMC9269970; doi:10.1371/journal.pone.0260173)
Supplement: Attachment — Submitted filename: Response to Reviewers.docx [file pone.0260173.s007.docx]

Response to reviewers:

We want to thank the reviewers for their effort and input that motivated us and gave us additional ideas and resulted in a manuscript that in our opinion is now considerably improved.

Reviewer 1:

Reviewer 1 raised several interesting points that we tried to address as thoroughly as possible.

Point 1: Have the authors tested the direct addition method in other semi-adherent cell lines?

Following up on the reviewer’s question, we have tested the same conditions on PC-12 cells that are growing semi-adherently and added two figures in the supporting information section. The images show that pre-coating with either PDL or PEI strongly increases adherence of these cells, however, only addition of PEI results in strong adherence but not that of PDL. Thus, for direct addition, the working concentration of PEI or PDL might have to be determined individually for each semi-adherent cell line. Text dealing with this topic has been added to the manuscript.

Point 2: Whether pre-coated with other cationic polymer (e.g., PEI) or using direct addition method or under different coating/addition conditions would impact the correlation between the cell density and bioluminescence? Any data to support?

To address this question, we changed Figure 1 to show in a direct comparison the effect of pre-coating or lack thereof on the bioluminescence signal. There is apparently a trend for stronger bioluminescence in untreated wells as compared to pre-coated ones that however is not significant. Moreover, as figure 3 indicates, there is no difference between the bioluminescence measured in cells with low adherence vs. that measured in strongly attached cells that can withstand the rinsing challenge.

Point 3:

In figure 3, is there any explanation on why the rinsed conditions in the optimum concentration range for both cationic polymers, especially PDL, are consistently higher than control?
We noticed this of course, too. However, as the data for rinsed in question were not significantly different (multiple t-test) from the respective data for controls, we did not consider this as relevant. Indeed, in most of the other experiments it's the other way around. It could be an edge effect, because the control cells were located in row A that sometimes has a somewhat lower cell density despite have received the same amounts of cells (at least in theory). For following experiments we alternated.

Point 4:Is there any further investigation into the protective effects of FBS in direct addition method (e.g., contributing components and minimal FBS concentration required)?

This is an interesting question, too, but was not the main topic of this study. One might speculate that as higher concentrations of PDL or PEI are harmful to the cells even in the presence of FBS, that without FBS lower concentrations of PDL or PEI should be used and might be enough to promote cell adherence. We tested whether in the absence of FBS lower amounts of PDL or PEI would allow the cells to grow healthy and still well attached. As this however, disappointingly, was not the case (with lower concentrations of PDL/PEI the cells were not well attached) we did not continue in that direction.

Reviewer 2:

We are sorry that we obviously gave reviewer 2 a hard time with reading our manuscript. In the revised version we tried to improve its legibility by rephrasing significant parts of it and addressing the concerns raised in this thorough and detailed review. We want to thank him for pointing out that this topic is of high importance to the veterinary field as we were not aware of this and actually would like to know more about it.

General response:

Introduction:

We entered the full name of the HEK293 cells and stated the aim of our study in more detail

Materials and methods:

We enter the lacking references, corrected inaccuracies and tried to describe the procedures in a less confusing way, giving more information and explaining the methods in more detail (in part also entering relating details in the supporting material section).

Line 103:

The source of our cells was given in line 102 and 104. The cells were checked by vision for health and viability. A mycoplasma test was once performed and found negative.

As hardly any cell were floating the next day after seeding under normal conditions it was assumed that they are in principle all viable. Of course, there were experiments with cells that did not look healthy e.g. when plated without FBS in the presence of PDL or PEI. But these are conditions that are not to be recommended anyway.

Results:

We hope that with the changes and additions in the “Materials and methods” as well with the new supporting information it is now more evident how we designed and performed our experiments.

More detailed responses:

Line 161 “2-3 days”. The time should be defined or 2 or 3 days, better 48 or 72 hours. The evaluation should be performed at 24 or 72 hours, with no margin of approximation, as cell growth and cell adhesion can vary greatly within this time frame.

Indeed, the cells are not always growing the same way that’s why we gave this time frame of 2-3 days. The effect e.g. of different lots of FBS is not negligible. But the fact that despite this the STDs are acceptable demonstrates the robustness of the results and the assay.

Line 286-287 How did the authors calculate the % of cells to seed in each well? How did the authors say that 50000 is “a lower one that under normal circumstances” and 80000 is “a higher one that…”. In legend of Fig 1 the authors write that they seeded 30000 cells/96 wells. Is it possible to have a justification fo the choice of these numbers?

This included various experiments with different non-standardized numbers of cells. From these experiments we learned that it takes more than 80.000 cells/well for the next day to be confluent, otherwise they are subconfluent. For the experiments with a measurement after 2-3 days in general approximately 30.000 cells/well were used as described to obtain confluent monolayers after 2-3 days.

Line 321 Therefore? What could be the actual usefulness and feasibility of precoating a plate with cationic polymers in relation to the non use of SFB? The authors stae that in any case, serum is needed for cells growing. Would not it have been more useful to test with an artificial serum? In this way, it could have been said that system can be considered as animal-free model.

As written in the manuscript the pre-coating without FBS in the medium afterwards was intended as a negative control that turned out to give an unexpected result.

Following the suggestion of the reviewer we added new, in our opinion interesting experiments in the supporting informations with animal-free medium where the FBS is substituted with human platelet lysate.

Figure and graphics.
The graphics are not clear: symbols overlap and it is very difficult to have a clear view of the data (fig 3A and B, fig 4 A and B).

We reduced the symbol sizes and use now one-sided SEM-bars.

Fig 4, the legend is not clear. Fig 5 is not clear, there are too much data and information to link each other.

Fig 4 We tried to make the legend more intelligible

Fig. 5 We changed the figure design and added color
